# Establishment of High-Efficiency Screening System for Gene Deletion in *Fusarium venenatum* TB01

**DOI:** 10.3390/jof8020169

**Published:** 2022-02-10

**Authors:** Sheng Tong, Kexin An, Wenyuan Zhou, Wuxi Chen, Yuanxia Sun, Qinhong Wang, Demao Li

**Affiliations:** 1 Tianjin Key Laboratory for Industrial Biological Systems and Bioprocessing Engineering, Tianjin Institute of Industrial Biotechnology, Chinese Academy of Sciences, Tianjin 300308, China; tongs@tib.cas.cn (S.T.); ankx@tib.cas.cn (K.A.); zhouwy@tib.cas.cn (W.Z.); chen_wx@tib.cas.cn (W.C.); sun_yx@tib.cas.cn (Y.S.); wang_qh@tib.cas.cn (Q.W.); 2 National Center of Technology Innovation for Synthetic Biology, Tianjin 300308, China

**Keywords:** *Fusarium venenatum*, homologous recombination rate, screening efficiency, visualized gene deletion system, fluorescence observation, *ku70*

## Abstract

Genetic engineering is one of the most effective methods to obtain fungus strains with desirable traits. However, in some filamentous fungi, targeted gene deletion transformant screening on primary transformation plates is time-consuming and laborious due to a relatively low rate of homologous recombination. A strategy that compensates for the low recombination rate by improving screening efficiency was performed in *F. venenatum* TB01. In this study, the visualized gene deletion system that could easily distinguish the fluorescent randomly inserted and nonfluorescent putative deletion transformants using green fluorescence protein (GFP) as the marker and a hand-held lamp as the tool was developed. Compared to direct polymerase chain reaction (PCR) screening, the screening efficiency of gene deletion transformants in this system was increased approximately fourfold. The visualized gene deletion system developed here provides a viable method with convenience, high efficiency, and low cost for reaping gene deletion transformants from species with low recombination rates.

## 1. Introduction

*F. venenatum*, identified from more than 3000 soil organism samples from across the world, has been successfully used as a food-based cell factory [1,2,3]. The mycoprotein produced by it offers a better nutritional balance than animal- and plant-derived protein, including a reduction in saturated fat, an increase in dietary fiber, and richness in essential amino acids [4]. Compared to the single-cell protein, which is based on the cultivation of protein-producing microorganisms, including bacteria, yeasts, and microalgae, the mycoprotein is more flavorful and has a meat-like texture [2,5]. Moreover, the mycoprotein, as “generally recognized as safe” (GRAS), was accepted by the U.S. Food and Drug Administration (FDA) and has been sold lawfully in more than 10 countries since 1985 [2,6,7]. Therefore, it has the potential to partially or entirely substitute animal- and plant-derived protein foods that are facing insufficient protein supply due to the aggravation of population growth and environmental pollution [8,9,10].

Genetic engineering is one of the most effective methods to obtain strains with desirable traits. Currently, many genetic transformation systems have been reported in filamentous fungi, such as *Beauveria bassiana*, *Myceliophthora thermophila*, *Trichoderma reesei*, and *Aspergillus niger*, and *Agrobacterium-tumefaciens*-mediated transformation (ATMT) and PEG-mediated protoplast transformation are the dominant methods [11,12,13,14,15,16]. Gene knockout is one of the most effective methods to study gene function at the gene level. Generally, the knockout cassette integrates into the genome either randomly or specifically, and the homologous recombination rate varies greatly from species to species [17]. In *F. venenatum*, both of the abovementioned transformation systems have been reported; however, the transformation efficiency and homologous recombination frequency were relatively low, which has hindered the development of high-throughput gene knockout procedures [17,18]. Therefore, methods for improving the homologous recombination rate and screening efficiency are urgently needed.

Some studies have reported that the selection of resistance genes, the length of homology arms, and the form of donor DNA (linear/circular plasmid or PCR product) had certain effects on the homologous recombination frequency [19,20]. Specifically, disruption of *ku70*, which is required for the nonhomologous end joining (NHEJ) pathway of DNA repair [21], dramatically increases the homologous recombination frequency with short homologous flanks in filamentous fungi, including *Neurospora crassa*, *Aspergillus niger*, *Trichoderma reesei*, and *Myceliophthora thermophila* [16,20,22,23,24]. However, to date, the function of the *ku70* gene in *F. venenatum* TB01 has not yet been tested. Additionally, some visual screening systems based on fluorescent reporter genes or pigment-coding genes have been successfully applied to filamentous fungi, such as *Myceliophthora thermophila* and *Aspergillus niger*, to improve screening efficiency [16,25,26]. However, again, similar studies have not been reported in *F. venenatum* TB01.

In this study, we developed a PEG-mediated protoplast transformation system in *F. venenatum* TB01, and gene overexpression or knockout could be accomplished successfully despite a low rate of homologous integration. To further improve the homologous recombination rate, we tried to knock out the *ku70* gene in *F. venenatum* TB01, but ultimately failed. Therefore, a visualized gene deletion system using green fluorescence protein (GFP) as the marker and a hand-held lamp as the tool was developed to improve screening efficiency for targeted gene deletion. The chitin synthase gene (*Chs*) disruption experiment indicated that fluorescent randomly inserted transformants can be easily filtered out in a few seconds, resulting in an approximately fourfold increase in the screening efficiency of gene deletion transformants in this system compared to the conventional method, in which each transformant needed to be verified by direct PCR. These data demonstrated that this visualized gene deletion system is feasible and has the potential to be applied to species with low homologous recombination rates.

## 2. Materials and Methods

### 2.1. Preparation of F. venenatum and E. coli DH5α Cultures for Transformation

*F. venetianum* TB01 was isolated by the Tianjin Institute of Industrial Biotechnology and deposited at CGMCC under accession number CGMCC NO. 20740. The organism and its derived strains were conserved as a mixture of dry conidia at −80 °C and routinely grown in/on glucose yeast extract broth/agar (GYB/GYA) at 28 °C. *E. coli* DH5α used for gene cloning and vector construction was grown in/on LB broth/agar at 37 °C. The formulations of the medium above are listed in Supplementary Table S1.

### 2.2. Quantification of Conidia Yield

Quantification of conidia yield was performed as described [27] with some modifications. Briefly, 60 μL conidial suspensions (5 × 10^6^ conidia/mL) derived from GYA plates were spread onto GYA, CMC-Na, PDA (Coolaber, Beijing, China), CZA, and 1/2 SDA plates (90-mm-diameter, Twbio, Beijing, China), respectively. After incubation at 28 °C for 10 days, conidia on each plate were collected in 3 mL Tween 80 (0.05% (vol/vol)) and filtered through four layers of lens-cleaning paper to remove mycelial debris. The number of conidia produced was counted using a hemocytometer under a microscope (Olympus CX43, Tokyo, Japan). The experiments were repeated three times with three replicates for each culture medium. The formulations of the medium above are listed in Supplementary Table S1.

### 2.3. Antibiotic Sensivity Test for F. venenatum

Two microliters of conidial suspensions (5 × 10^6^ conidia/mL) derived from GYA plates were spread onto the center of GYA plates (60-mm diameter, Twbio, Beijing, China) containing geneticin (Sangon, Shanghai, China) or hygromycin (Sangon, Shanghai, China) at the indicated concentration (0–50 μg/mL). After 3 days of incubation at 28 °C, representative images were taken.

### 2.4. Construction of Targeted Gene Knockout and eGFP Gene Overexpression Vector

For construction of the targeted gene deletion vector, the geneticin resistance gene (*neo*) cassette (~1.9 kb) was used to replace a ~0.9–1.1 kb gene fragment containing ~0.5 kb of the 5′-terminus of the targeted gene. The upstream and downstream fragments (~1.6 kp) of *Chs* (FVRRES_06397), putative *Azf* (FVRRES_12147), and *ku70* (FVRRES_08939) deletion vector were amplified via PCR with primer pairs PChsLB1/PChsLB2 and PChsRB1/PChsRB2, PAzfLB1/PAzfLB2 and PAzfRB1/PAzfRB2, and PKuLB1/PKuLB2 and PKuRB1/PKuRB2, respectively, using *F. venetinum* TB01 genomic DNA as the template and cloned into the plasmid vector pK2-*neo* (Supplementary Figure S1A). To construct the visualized gene deletion system using *Chs* as a test gene, the knockout cassette was amplified from pK2-Chs_upstream_-neo-Chs_downstream_ via PCR with primer pairs PChsLB3/PChsRB2 and cloned into the site between *Pme*I and *Xba*I of pK2-*gpdA::GFP-neo* (Supplementary Figure S1B). For construction of the e*GFP* overexpression vectors, the fragment containing the ORF of e*GFP* and the terminator Ttrpc, and promoter sequences of *gpdA* (FVRRES_09878), *gla* (FVRRES_01380), and *tef* (FVRRES_13282) were amplified with the corresponding primers. The obtained *GFP*-Ttrpc and promoter fragment were cloned into the *Eco*RI site of the pK2-*neo* vector by seamless cloning (Vazyme, Nanjing, China). The insertion cassette or knockout cassette used for protoplast transformation was finally amplified from the constructed vector with primer pairs PpKLB1/PpKLB2 located in the vector backbone. The primers used above are listed in Supplementary Table S2.

### 2.5. PEG-Mediated Protoplast Transformation

The preparation of protoplasts was conducted as previously described [18] with some modifications. Conidial suspensions (500 μL, 5 × 10^6^ conidia/mL) derived from GYA plates were cultured in 50 mL YEPD liquid medium at 28 °C and 180 rpm for 16 h. After centrifugation at 13,000× *g* for 15 min, the fungal germlings were resuspended in 0.7 M NaCl and washed twice. Then, the germlings were treated with 2 mg/mL driselase (Lablead, Beijing, China), 4 mg/mL smailase (Sangon, Shanghai, China), or a mixture of both suspended in 0.7 M NaCl at different time points at 32 °C, 130 rpm. Fungal protoplasts were collected by filtration with four layers of lens-cleaning paper and washed twice with STC buffer. The images of protoplasts were visualized using microscopy (Olympus CX43, Tokyo, Japan) after incubation on an enzyme mix over the indicated time course.

The transformation was carried out as previously described [18] with some modifications. Briefly, 100 μL of protoplast suspensions was mixed with 25 μL SPTC and 10 μg transforming PCR cassette in a 2 mL Eppendorf tube and incubated on ice for 40 min. Then, 1 mL SPTC was gently added to the above sample and incubated at room temperature for 30 min. After that, the mixture was mixed with regeneration medium (RM) preheated to approximately 42 °C and poured into 90-mm-diameter petri dishes. After overnight incubation, the selective medium containing 15 μg/mL geneticin was covered. Mycelium of *F. venenatum* were visible after 2–3 days of incubation at 28 °C.

### 2.6. Fluorescence of Fungus Strains

Site-specific (no fluorescence) or ectopic integration (fluorescence) colony transformants on plates were distinguished using a hand-held fluorescent protein excitation light source (LUYOR-3415GR, Shanghai, China). The transformation plates or 24-well plates inoculated with hyphae were placed in a dark environment; then, the green fluorescent excitation light source with excitation and emission wavelengths of 450 nm and 500 nm, respectively, was turned on to illuminate the plate and the colony transformants were observed or photographed through the filter. The mean fluorescence intensity of fluorescent strains cultured on GYA for 3 days (5 transformants/each promoter type) was quantified using ImageJ software [28]. The fluorescence in *F. venetinum* TB01 hyphae and conidia was detected by fluorescence microscopy (Leica DM5000B, Wetzlar, Germany) with excitation and emission wavelengths of 488 nm and 533 nm after culturing on GYA for 10 days.

### 2.7. Validation of the Transformants by PCR

The simple templates used for PCR analysis were prepared as described [29]. Briefly, a small amount of mycelia was picked from the colony into 8 μL 0.3 M NaOH. After incubating at 98 °C for 2 min, 160 μL 20 mM Tris-HCl (pH 8.0) and 8 μL 0.3 M HCl were added to neutralize the above solution, and simple templates containing fungal genomic DNA were completed. The PCR procedure was set according to the instructions of 2× Rapid Taq Master Mix (Vazyme, Nanjing, China), and the primers used in this study are listed in Supplementary Table S2.

## 3. Results

### 3.1. Optimization of the Protocol for Protoplast Transformation

**Figure 1:**
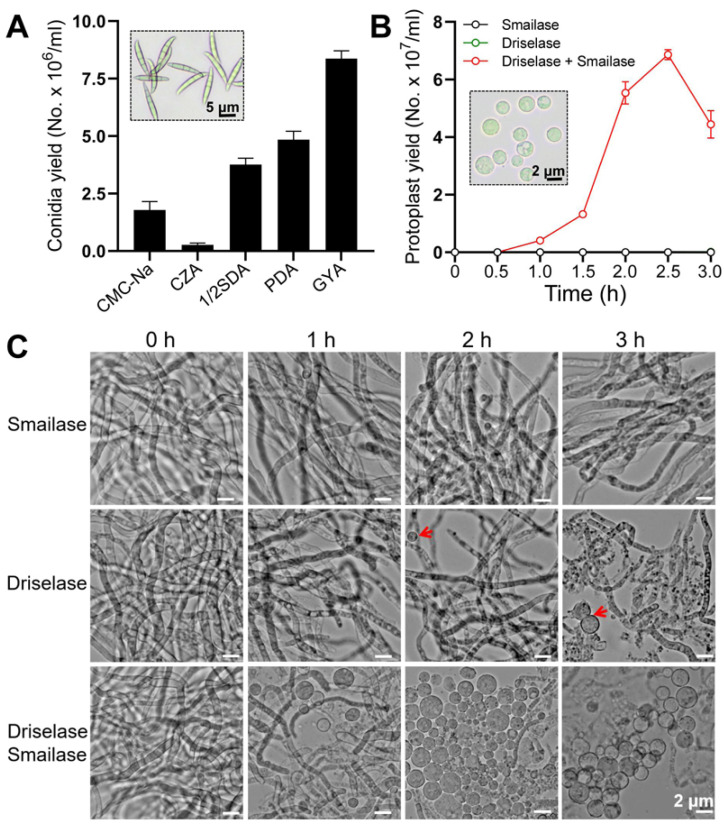
Quantification of conidial yield (**A**) and protoplast yield (**B**). Representative images of conidia or protoplasts are shown in dotted rectangular boxes. (**C**) Representative images of protoplasts treated with smailase, driselase, or a mixture of both at different time points. The concentrations of driselase and smailase were 2 mg/mL and 4 mg/mL, respectively. The protoplasts in the driselase treatment are indicated with red arrows.

Young mycelia germinated from conidia are the best material for preparing protoplasts [30]. Therefore, the culture medium for efficient conidia production needs to be determined first. The comparison results showed that *F. venenatum* TB01 cultured on GYA exhibited a higher conidial yield (8.4 × 10^6^/mL) than other media (Figure 1A). Then, protoplasts were prepared from young mycelium treated with driselase, smailase, or a mixture of both. Compared to driselase or smailase alone (almost no protoplasts), the germlings treated with the mixture produced a large number of protoplasts, and the maximum yield (6.9 × 10^7^/mL) was reached when incubated on the enzyme mix for 2.5 h (Figure 1B,C).

**Figure 2:**
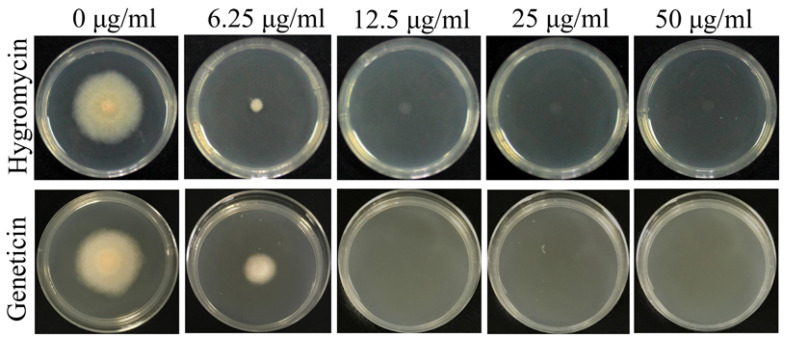
The sensitivity of *F. venenatum* TB01 to hygromycin and geneticin. Both antibiotics showed complete inhibition of *F. venenatum* TB01 at more than 12.5 μg/mL.

To confirm the appropriate antibiotic concentration for screening transformants, the sensitivity of this fungus to hygromycin and geneticin was tested. After incubation at 28 °C for 3 days, both antibiotics showed complete inhibition of *F. venenatum* TB01 at more than 12.5 μg/mL. Thus, the *hpt* gene conferring fungal resistance to hygromycin and the *neo* gene conferring fungal resistance to geneticin can both be employed as the selection marker for *F. venenatum* TB01 transformation (Figure 2).

### 3.2. Rate of Homologus Recombination in F. venenatum TB01

**Figure 3:**
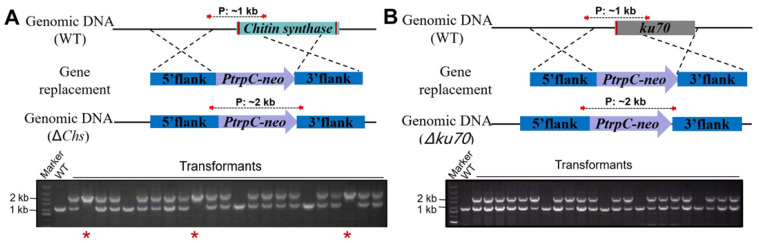
PCR analysis of *Chs* (**A**) and *ku70* (**B**) deletion transformants. The primers located in the 5′ flank and 3′ flank of the target gene knockout region are shown by red arrows, and the predicted sizes of PCR products in the wild type (~1 kb) and gene knockout mutant (~2 kb) are shown by dashed lines. The ectopic insertion mutant contained both bands (~1 kb and ~2 kb). Electrophoresis images displayed partial results of transformant screening. The confirmed Δ*Chs* mutants are indicated with red asterisks. Red rectangles in genomic DNA indicate the intron regions of the target gene.

Initially, the homologous recombination rate of *F. venenatum* TB01 was evaluated using *Chs* as a test gene. To distinguish false-positive (band size: ~1 kb), ectopic integration (band size: ~1 kb and ~2 kb) and gene knockout (band size: ~2 kb) transformants, a PCR primer pair located in the 5′ flank and 3′ flank of the target knockout fragment was designed. PCR analysis showed that the *Chs* gene was properly replaced by the neo cassette in 16.7% transformants (12/72), while randomly inserted transformants accounted for 72.2% (52/72) (Figure 3A). Thereafter, one randomly inserted and two *Chs* deletion mutants selected from transformants above were further confirmed by three separate primer pairs (Supplementary Figure S2). To ensure the authenticity of this rate, another gene, *Azf,* was also tested, and the homologous recombination rate (19.4%, 14/72) was similar to that of the *Chs* gene (Supplementary Figure S3). The above results indicated that the homologous recombination rate was relatively low in *F. venenatum* TB01. To improve gene knockout efficiency, we tried to disrupt the *ku70* gene. Regrettably, all tested transformants (~200) verified by two different primer pairs were randomly inserted (Figure 3B and Supplementary Figure S4), despite protoplast transformation being performed many times by three individuals.

### 3.3. Screening the Strong Promoter for the Visualized Gene Deletion System

**Figure 4:**
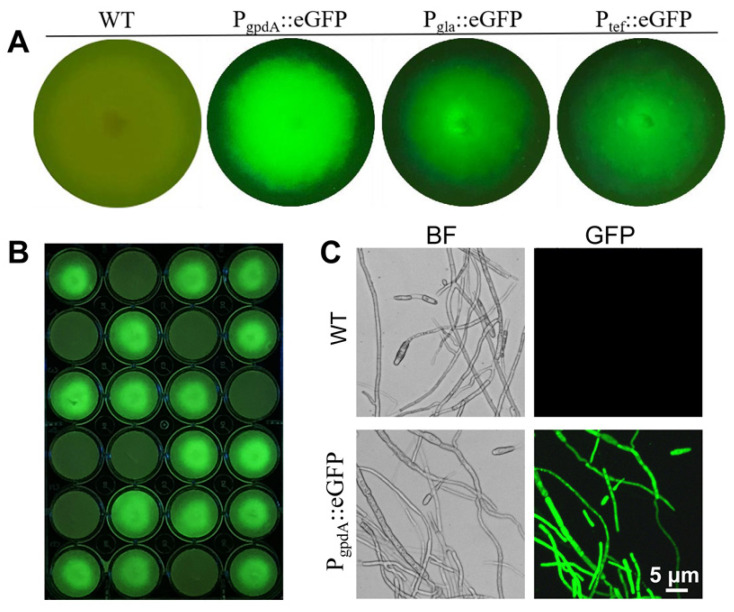
Screening of endogenous promoters with strong expression activity in *F. venenatum* TB01. (**A**) Representative images of fluorescent colonies. The *eGFP* reporter gene in *F. venenatum* TB01 was driven by endogenous promoters of *gpdA* (glyceraldehyde-3-phosphate dehydrogenase gene), *gla* (glucoamylase gene), and *tef* (translation elongation factor gene). (**B**) Fluorescence image acquisition of *PgpdA::eGFP*-expressing colonies on a 24-well plate using a hand-held lamp. (**C**) Representative images of *PgpdA::eGFP* expression in hyphae and conidia.

Changing a traditional viewpoint, the low knockout rate might be compensated to some degree by improving screening efficiency. Therefore, we planned to design a simple visual screening system using green fluorescence protein (GFP) as a marker for selection under a hand-held fluorescent protein excitation light source. The priority was to obtain a strong promoter. To identify suitable endogenous promoters, an e*GFP* reporter gene driven by the endogenous promoters PgpdA, Pgla, and Ptef was transformed into *F. venenatum* TB01 and evaluated. The results of fluorescence observation and quantification showed that *PgpdA::eGFP* conferred the strain with a strong fluorescence signal, while strains carrying the *Pgla::eGFP* and *Ptef::eGFP* constructs exhibited weak fluorescence signal (Figure 4A), and the mean fluorescence intensity in *PgpdA::eGFP* strains was approximately 3.5-fold higher than that in the strains carrying the other promoter constructs (Supplementary Figure S5). Additionally, statistical analysis showed that 67% of transformants (16/24) of *PgpdA::eGFP* strains showed fluorescence filled with mycelia and conidia under a fluorescence microscope (Figure 4B,C). Based on the above results, *PgpdA* was subsequently selected to build a visualized gene deletion system.

### 3.4. Visualized Gene Deletion System Significantly Improves the Screening Efficiency of Gene Deletion Transformants

**Figure 5:**
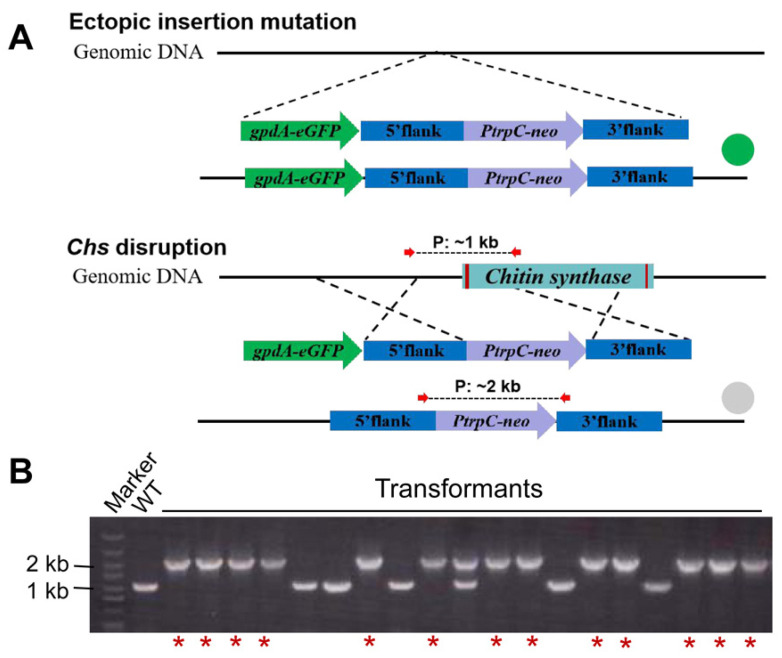
Evaluation of the visualized gene deletion system using *Chs* as a test gene. (**A**) Schematic diagram of the *Chs* knockout cassette integrating into the genome of *F. venenatum* TB01 via ectopic insertion (fluorescence) or homologous recombination (no fluorescence). (**B**) Diagnostic PCR was performed to confirm *Chs* deletion. The primers located in the 5′ flank and 3′ flank of the *Chs* knockout region are shown by red arrows, and the predicted sizes of PCR products in the wild type (~1 kb) and Δ*Chs* mutant (~2 kb) are shown by dashed lines. The ectopic insertion mutant contained both bands (~1 kb and ~2 kb). The confirmed Δ*Chs* mutants are indicated with red asterisks. Red rectangles in genomic DNA indicate the intron regions of the target gene.

In this system, the GFP-encoding cassette driven by PgpdA was incorporated into the *Chs* gene knockout cassette, which allowed for ectopic (fluorescence) or site-specific (no fluorescence) integration (Figure 5A). Based on this strategy, the *Chs* disruption experiment was performed again. After primary screening using the hand-held fluorescent protein excitation light source, the ectopic insertion mutations with obvious fluorescent signals (53/72) were filtered out, and then the nonfluorescent putative *Chs* deletion transformants (19/72) were further confirmed by PCR with the previous primer pair. The results showed that the *Chs* gene was knocked out in 13 of these 19 transformants (68.4%) (Figure 5B), indicating that the screening efficiency of gene deletion transformants in the visualized gene deletion system was approximately fourfold higher than that in the conventional screening method (direct PCR screening, 16.7%).

## 4. Discussion

**Figure 6:**
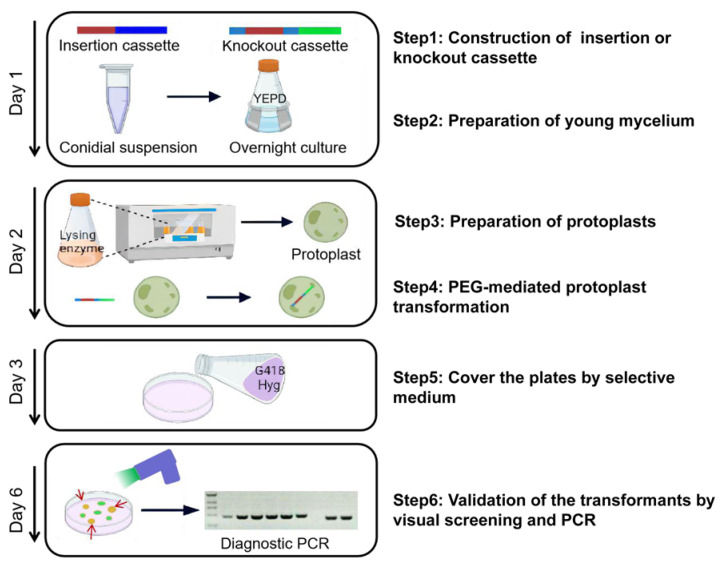
Flowchart of the visualized gene deletion system. This system is based on PEG-mediated protoplast transformation and fluorescence visualization. The insertion cassette or knockout cassette was amplified from the constructed vector. The conidia were cultured overnight to obtain fresh mycelium and then made into protoplasts by lysing enzymes. After that, the targeted cassette was transformed into protoplasts by PEG-mediated transformation. The primary transformation plates were covered with selective medium containing the appropriate concentration of antibiotics the next day. Finally, the candidate plates were illuminated for 3 s using a hand-held lamp, and nonfluorescent putative deletion transformants were further confirmed by PCR. The entire process could be accomplished successfully within a week.

Genetic transformation systems have been established in a large number of filamentous fungi [14,15,31,32,33,34,35]; however, the homologous recombination rate varies greatly from species to species [17]. In the present study, the entire process of gene overexpression or knockout from cassette construction to transformant validation could be accomplished successfully within a week by PEG-mediated protoplast transformation for *F. venenatum* TB01, and the flowchart is shown in Figure 6. The results of gene knockout showed that the homologous recombination rate (15–20%) in *F. venenatum* TB01 was in the range of that reported in *F. venenatum* ATCC 20334 (main range from 5% to 50%) [19]. To increase the recombination frequency, the *ku70* gene, which is required for the NHEJ pathway of DNA repair, was selected for deletion. Interestingly, the knockout ultimately failed, despite protoplast transformation being performed many times by three individuals (Figure 3B and Supplementary Figure S4), suggesting a mechanism for self-protection by blocking *ku70* gene deletion or death caused by *ku70* gene deletion.

The strong promoter is a key factor in developing the visualized gene deletion system for *Fusarium venenatum* TB01. Fluorescence observation revealed that the e*GFP* reporter gene driven by endogenous promoter PgpdA conferred the strain with the strongest fluorescence signal (Figure 4), which was similar to a variety of other fungi [36,37,38]. In the visualized gene deletion system, the candidate plates just need to be illuminated a few seconds using the hand-held fluorescent protein excitation light source, then the fluorescent randomly inserted and nonfluorescent putative deletion transformants can be easily distinguished at a glance. Therefore, compared to the microscope-based screening [16], the visual screening based on the hand-held lamp has the advantages of convenience, speediness (putative deletion transformants can be screened out within 5 s), and high efficiency (more than 150 transformants can be distinguished per minute). When performing the *Chs* disruption experiment, all fluorescent transformants (53/72) were filtered out after primary screening, and the nonfluorescent putative deletion transformants (19/72) were further confirmed by PCR. The electrophoresis result revealed that a randomly inserted transformant with two PCR products was found among the nonfluorescent putative deletion transformants (Figure 5B). We speculated that the genomic integration site of the cassette weakened the expression of eGFP driven by PgpdA in this transformant and resulted in invisible fluorescence under the hand-held lamp. Additionally, the visual screening system increased the screening efficiency for targeted gene deletion by ~fourfold; however, in fact, the homologous recombination rate was hardly changed compared with that in the conventional screening method. To improve the efficiency of gene knockout or deletion in essence, an efficient gene editing system, such as CRISPR-Cas9, which has been developed recently in *F. venenatum* A3/5 [39], could be adapted for *Fusarium venenatum* TB01. Coincidentally, the PgpdA::eGFP strain and the visualized gene deletion system can provide available edit object and screening approaches for the establishment of gene editing systems in the future. Some studies have reported that the length of the homology arm could contribute to homologous recombination frequency [20,24]. In this study, the length of the homology arm (~1.6 kb) was similar to what has been reported in *F. venenatum* ATCC 20334 (~1.5–1.7 kb) [19], and we hypothesized that the efficiency of gene knockout might be further improved by increasing the length of homology arms. Furthermore, *Agrobacterium-tumefaciens*-mediated transformation deserves establishment in future work to evaluate the homologous recombination frequency in *Fusarium venenatum* TB01.

## Data Availability

The data presented in this study are available in this manuscript and supplementary material and can be requested from the corresponding author.

## Supplementary Materials

The following supporting information can be downloaded at: https://www.mdpi.com/article/10.3390/jof8020169/s1. Table S1. The formulations of medium and reagent in this study. Table S2. Primers used in this study. Figure S1. Vector map of pK2-*neo* (A) and pK2-gpdA::GFP-*neo* (B). Figure S2. PCR analysis of *Chs* deletion transformants. Figure S3. PCR analysis of *Azf* deletion transformants. Figure S4. PCR analysis of *ku70* deletion transformants. Figure S5. Quantification of the mean fluorescence intensity of fluorescent strains by ImageJ software.
