# Supplementary material for: Establishment of High-Efficiency Screening System for Gene Deletion in Fusarium venenatum TB01
Source: J Fungi (Basel). 2022 Feb 10;8(2):169. doi: 10.3390/jof8020169 (PMC8878023; doi:10.3390/jof8020169)
Supplement: Supplementary file 1 [file jof-08-00169-s001.zip › jof-1577049-supplementary.pdf]

Journal of Fungi

MDPI

Article

Supplementary Materials: Establishment of High-Efficiency Screening System for Gene Deletion in *Fusarium venenatum* TB01

Sheng Tong [1,2], Kexin An [1,2], Wenyuan Zhou [1,2], Wuxi Chen [1,2], Yuanxia Sun [1,2], Qinhong Wang [1,2] and Demao Li [1,2,*]

Table S1. The formulations of medium and reagent in this study.

Name	Formulation（L）	Use
LB	NaCl 10 g, Yeast extract 5 g, Tryptone 10 g	Cultivation of *E. coli*
GYB	Glucose 20 g, Yeast extract 10 g	
CMC-Na	CMC-Na 10 g, NH_4SO_4 5 g, Yeast extract 1 g, $MgSO_4$ 1.5 g, KH_2PO_4 2 g, KCl 0.7 g, Na_2SO_4 0.5 g	
CZB	$NaNO_3$ 2 g, Sucrose 30 g, $MgSO_4{\cdot}7\ H_2O$ 0.5 g, $FeSO_4{\cdot}7\ H_2O$ 0.001 g, K_2HPO_4 1 g, KCl 0.5 g; pH 7.0	Cultivation of *F. venenatum*
1/2 SDB	Glucose 10 g, Tryptone 2.5 g, Yeast extract 5 g	
PDB	26 g powder	
YEPD	Yeast extract 3g, Tryptone 10g, Glucose 20g	
RM*	Yeast extract 1 g, Tryptone 1 g, Sucrose 274 g	Protoplast transformation of *F. venenatum*
STC	0.8 Msorbitol, 50 mM $CaCl_2$, 50 mM Tris-HCl; Ph 8.0	
SPTC	40 % PEG6000 in STC	

The solid medium requires 1.5% (w/v) agar. * Agarose (1% (w/v)) instead of agar.

Table S2. Primers used in this study.

Name	Sequence (5′ to 3′)	Use
PChsLB1	ACAGCTATGACCATGATTACGAATTCTGGTCGGTTGTGTCTGGTTC	Targeted gene knockout
PChsLB2	CAATGTCATCTTCTGTCGACACTAGTGGTTTGGTTGGTGTGGGAGTG	
PChsLB3	ACAGGTACACTTGTTTAGAGGCACTAGTTGGTCGGTTGTGTCTGGTTC	
PChsRB1	GTTTAGAGGTAATCCTTCTTTCTAGAGCTTTCGGTCATTATCCTCC	
PChsRB2	GCTTGCATGCCTGCAGGTCGACTCTAGAGAGCGAGAACCAACTGAACA	
PAzfLB1	ACAGCTATGACCATGATTACGAATTCCGGAAGTGGGGATACATCAG	
PAzfLB2	CAATGTCATCTTCTGTCGACACTAGTGAGTCAGGACGCAAAGAGACC	
PAzfRB1	GTTTAGAGGTAATCCTTCTTTCTAGATTCAGCGCAAGGCAGAGATG	
PAzfRB2	GCTTGCATGCCTGCAGGTCGACTCTAGAACTCTCGCTCCTCCATCTTC	
PKuLB1	ACAGCTATGACCATGATTACGAATTCGGTCTTAGTTGGAGTTGGTG	
PKuLB2	CAATGTCATCTTCTGTCGACACTAGTGGGATATCTACAAGCCAGCTC	
PKuRB1	GTTTAGAGGTAATCCTTCTTTCTAGAGTGAAGACGTCAAGGCTCTA	

PKuRB2	GCTTGCATGCCTGCAGGTCGACTCTAGATATGCCTAACACTCCGCTCA	
PChsyz1	CACTCCCACACCAACCAAAC	
PChsyz2	GGTCAGGAGGATAATGACCG	
PChsyz3	TATCTGGCGCTCTGTATGAACG	
PChsyz4	AGTTCGGTTTCAGGCAGGTC	
PChsyz5	CTGTCGGGCGTACACAAATC	
PChsyz6	CCATGGTACCCGGATAGAAC	
PChsyz7	GCACCAGCCCACAATCCTTT	Transformants screening
PChsyz8	TCGACTCGTTTTGTTCGGGG	
PAzfyz1	GGTCTCTTTGCGTCCTGACT	
PAzfyz2	CATCTCTGCCTTGCGCTGAA	
PKuyz1	GAGCTGGCTTGTAGATATCC	
PKuyz2	TAGAGCCTTGACGTCTTCAC	
PKuyz3	GGACACTACGGTATTCCAAG	
PKuyz4	AATGTCCTCGTTCCTGTCTGC	
PgpdA1	TATGACCATGATTACGAATTCGGTGTTGATCGTCAACCAAGTCC	
PgpdA2	CACCATGGTGGCGACGAATTTTTGTTAAGGAGGTTCTGTTTGAGGAAA-GAT	
Pgla1	TATGACCATGATTACGAATTGAATACTT-GTTTCAGCGTTACAATTTCCTTTTGA	
Pgla2	CACCATGGTGGCGACGAATTTTTTATTTTTTTTTGCTTTTTCTAAATTT-GATTGTATATCCTAAGGT	eGFP overexpression
Ptef1	TATGACCATGATTACGAATTCCACCCGTGGGGAGCTGTAT	
Ptef2	CACCATGGTGGCGACGAATTATTGTTTAACTGGATCAGTCAAGTGG-TAGATAG	
Pgfp-T1	GTCGCCACCATGGTGAGCAA	
Pgfp-T2	CATCTTCTGTCGACACTAGTGCCTCTAAACAAGTGTACCTG	
PpKLB1	GGTGGCAGGATATATTGTGG	Amplification of cassette
PpKLB2	TTGGTTGGTCAAGTCCTGGT	

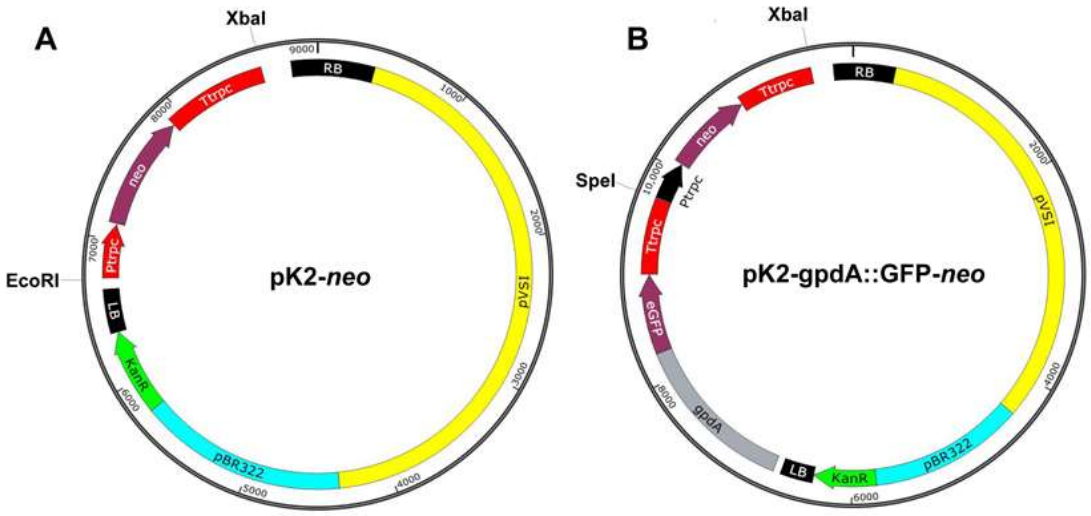

Figure S1. Vector map of pK2-*neo* (**A**) and pK2-gpdA::GFP-*neo* (**B**). The upstream fragment (5′ flank) of the target knockout region was cloned into the *Eco*RI site of pK2-*neo*; the downstream fragment (3′ flank) of the target knockout region was cloned into the *Xba*I site of pK2-*neo*; and the *Chs* knock-out cassette was cloned into the site between *Pme*I and *Xba*I of pK2-gpdA::GFP-*neo*.

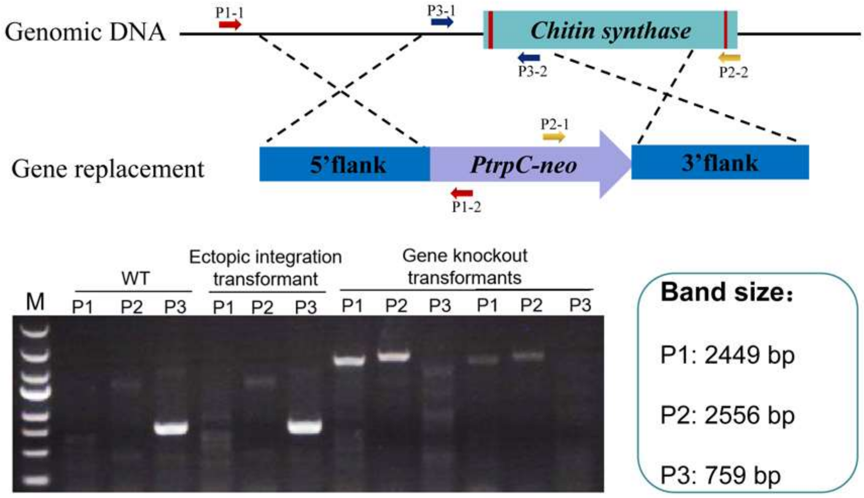

Figure S2. PCR analysis of *Chs* deletion transformants. The primer pairs are shown with arrows, and the predicted sizes of the PCR products are shown inside the rounded rectangle. P1-1 and P2-2 were located in the uptream of the 5′ flank and downtream of the 3′ flank in the genomic DNA, respectively; P1-2 and P2-1 were located in the *neo* gene cassette; P3-1 and P3-2 were located in the *Chs* knockout region. In the *Chs* deleting transformants, PCR bands of 2449 bp and 2556 bp were obtained by primer pairs P1 and P2, respectively. Red rectangles in genomic DNA indicate the intron regions of target gene.

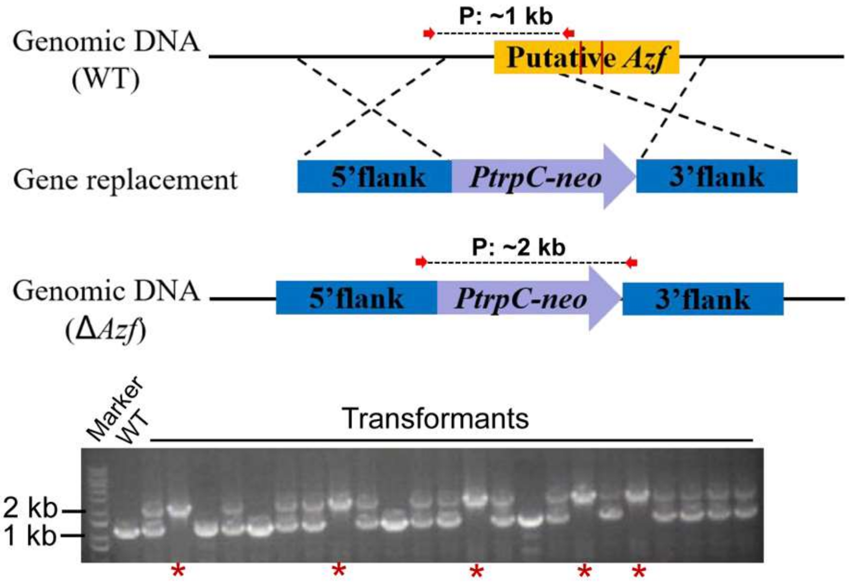

Figure S3. PCR analysis of *Azf* deletion transformants. The primers located in the 5′ flank and 3′ flank of the *Azf* knockout region are shown by red arrows, and the predicted sizes of PCR products in the wild type (~1 kb) and Δ*Azf* mutant (~2 kb) are shown by dashed lines. The ectopic insertion mutant contained both bands (~1 kb and ~2 kb). Electrophoresis images displayed partial results of transformant screening. The confirmed Δ*Azf* mutants are indicated with red asterisks. Red rectangles in genomic DNA indicate the intron regions of target gene.

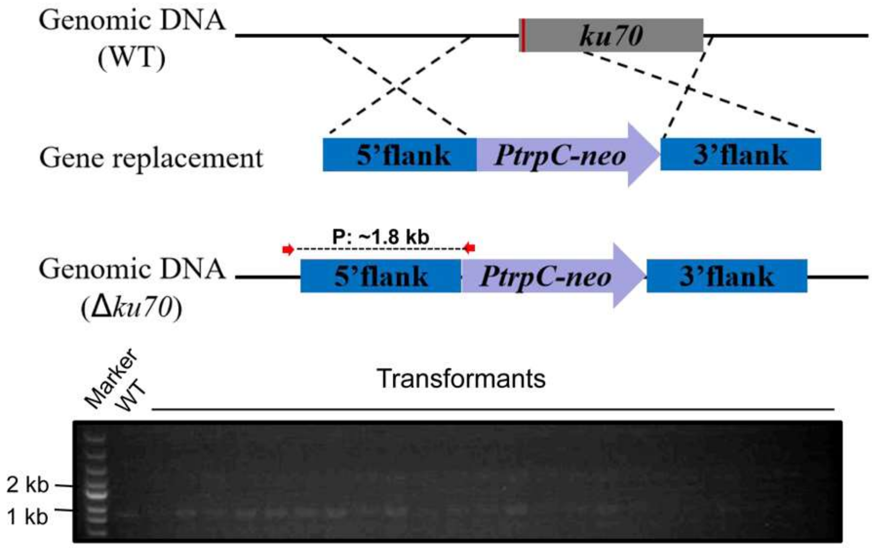

Figure S4. PCR analysis of *ku70* deletion transformants. The primer pair with one location in the *neo* gene cassette and the other location in the uptream of the 5′ flank in the genomic DNA for diagnostic PCR are shown by red arrows, and the predicted size of PCR product in the Δ*ku70* mutant (~1.8 kb) is shown by the dashed line. The results indicated that no *ku70* deletion transformant was identified. Electrophoresis images display partial results of transformant screening. Red rectangle in genomic DNA indicate the intron region of target gene.

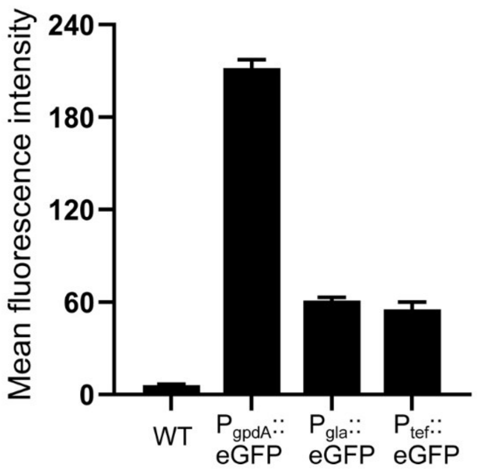

Figure S5. Quantification of the mean fluorescence intensity of fluorescent strains by ImageJ software. The *eGFP* reporter gene was driven by the endogenous promoters of *gpdA* (glyceraldehyde-3-phosphate dehydrogenase gene), *gla* (glucoamylase gene), and *tef* (translation elongation factor gene). Error bars = SD.
